# Tumor Necrosis Factor Receptor Associated Factors (TRAFs) 2 and 3 Form a Transcriptional Complex with Phosho-RNA Polymerase II and p65 in CD40 Ligand Activated Neuro2a Cells

**DOI:** 10.1007/s12035-016-9742-4

**Published:** 2016-02-03

**Authors:** Jimmy El Hokayem, George C. Brittain, Zafar Nawaz, John R. Bethea

**Affiliations:** 1 Department of Biochemistry and Molecular Biology, University of Miami, Miller School of Medicine, Miami, FL USA; 2 Neuroscience Program, University of Miami, Miller School of Medicine, Miami, FL USA; 3 The Miami Project to Cure Paralysis, University of Miami, Miller School of Medicine, Miami, FL USA; 4 Department of Biology, Drexel University, 3245 Chestnut Street, Philadelphia, PA 19104 USA

**Keywords:** CD40, NF‐kB transcription factor, Nucleus, TNF receptor associated factor (TRAF), Transcription, Neuron, Tumor necrosis factor (TNF)

## Abstract

The tumor necrosis factor receptor-associated factors (TRAFs) have been classically described as adaptor proteins that function as solely cytosolic signaling intermediates for the TNF receptor superfamily, Toll-like receptors (TLRs), NOD, like receptors (NLRs), cytokine receptors, and others. In this study, we show for the first time that TRAFs are present within the cytoplasm and nucleus of Neuro2a cells and primary cortical neurons, and that TRAF2 and TRAF3 translocate into the nucleus within minutes of CD40L stimulation. Analysis of the transcriptional regulatory potential of TRAFs by luciferase assay revealed that each of the TRAFs differentially functions as a transcriptional activator or repressor in a cell-specific manner. Interestingly, ChIP-qPCR data demonstrate that TRAFs 2/3, p65, and pRNAPol II form part of a transcriptional complex on the Icam-1 gene promoter upon CD40L stimulation. We further determined that TRAF2 recruitment to the nucleus is critical for the ubiquitination of H2b, a transcription permissive epigenetic modification. Our findings demonstrate for the first time that TRAFs 2/3 participate in the formation of a CD40L-induced transcriptional complex in neuronal cells.

**Electronic supplementary material:**

The online version of this article (doi:10.1007/s12035-016-9742-4) contains supplementary material, which is available to authorized users.

## Introduction

The tumor necrosis factor receptor-associated factors (TRAFs) have been classically described as cytosolic adaptor proteins that function as signaling intermediates for different receptor families, such as the TNF receptor superfamily, Toll-like receptors (TLRs), NOD, like receptors (NLRs), cytokine receptors, and others. TRAFs are thought to facilitate interactions between kinases and other signaling platforms and corresponding upstream receptors. Since the receptors that the TRAFs interact with lack catalytic activity of their own, activation of downstream signaling pathways has been hypothesized to be orchestrated predominantly by TRAF proteins, although the exact mechanisms by which the TRAFs regulate such activity remain to be elucidated ([1, 2] and references therein ).

There are currently seven known members of the TRAF protein family. Of these, TRAFs 1–6 were identified based upon the presence of a conserved C-terminal TRAF domain, which has been found to mediate the homo-/hetero-oligomerization of the TRAFs with one another, as well as interactions with the various upstream regulators and downstream effectors [3–8]. TRAFs 1–6 also contain a variable number of TRAF zinc-finger domains, and, with the exception of TRAF1, a RING finger domain [1] The RING finger domain is common to many E3 ubiquitin ligases, and in fact, TRAFs 2, 3, 5, and 6 have been reported to possess E3 ubiquitin ligase activity [2, 9–11]. TRAF7 was more recently identified to be a TRAF protein based upon the similarity of its RING- and zinc-finger domains to those of the TRAFs; however, it lacks the C-terminal TRAF domain [12].

While most recent studies describe TRAFs as cytosolic adaptor proteins with E3 ubiquitin ligase activity earlier studies compared TRAFs 1–3 to the STAT protein family, due to their sequence homology and the presence of both RING- and zinc-finger domains, which are traditionally considered to be DNA-binding domains [13–17]. In support of this possibility, there are several examples in which TRAFs have been found within the nucleus [18–20] and other instances where overexpression of either the full-length or domain-deletion isoforms explicitly altered the subcellular localization of TRAFs [20–22]. Furthermore, while nuclear localization sequences (NLSs) have not been identified for the TRAFs, TRAF3 has been found to interact directly with p62 nucleoporin, which binds to classical NLS-containing import complexes during activity-dependent nuclear translocation [23].

In our preliminary studies, we analyzed the intracellular activation of components of the nuclear factor-kappa B (NF-kB) signaling pathways downstream of the CD40 receptor in mouse cortical neurons and Neuro2a cells. We focused our studies on the CD40 receptor for two reasons: first, it is among the few receptors that are known to activate both the classical and the alternative NF-kB pathways. Second, it has been shown to interact with the majority of the TRAF proteins: TRAFs 1, 2, 3, 5, and 6 [5, 7, 8, 23, 24]. In addition, the CD40 receptor has been shown to be both present and functional in primary neurons [25]. Our preliminary data demonstrated the presence of TRAFs within the nucleus. Therefore, we hypothesized that the TRAFs may have a nuclear function and decided to investigate further.

In this study, we show that each of the TRAFs are present within the nucleus of Neuro2a cells, and that TRAFs 2 and 3 translocate into the nucleus of Neuro2a cells following 5 to 10 min of CD40L stimulation. We also show that TRAF2 translocates into the nucleus of primary cortical neurons, while TRAF3 is constitutively present within the nucleus at relatively high levels. Once in the nucleus, these proteins interact with chromatin and are able to bind to the NF-kB consensus element. Furthermore, both TRAFs 2 and 3 appear to be essential components of the NF-kB DNA-binding complexes, and each of the TRAFs 1–7 can function as a transcriptional regulator, either as a coactivator or corepressor. Additionally, we found that TRAF2 can function directly as a transcription factor and that its transcriptional regulatory potential depends upon the presence of an intact RING finger domain. Within the nucleus, TRAF2 and TRAF3 bind to the Icam-1 gene promoter in a complex containing p65 and pRNAPol II. We further show that a complex containing TRAF2 ubiquinitates H2b. This is the first demonstration that TRAFs function within the nucleus to regulate transcription and epigenetic modification of histones.

## Experimental Procedures

### Cell Culture

Neuro2a cells were cultured at 37 °C in 1:1 Opti-minimum essential medium (MEM)/Dulbecco’s modified Eagle’s medium (DMEM), supplemented with 5 % fetal bovine serum (FBS), 100 IU/mL penicillin/streptomycin (P/S), and 2 mM l-glutamine (l-glut) (Gibco) [19]. Daudi cells were cultured at 37 °C in RPMI 1640 (Gibco) supplemented with 10 % FBS, 100 IU/mL P/S, 2 mM l-glut, 55 μM 2-mercaptoethanol (Fisher Biotech) , 20 μg/mL gentamycin, and 1 mM Na-pyruvate (Gibco) according to the American Type Culture Collection protocol. HEK 293 cells were cultured at 37 °C in DMEM supplemented with 10 % FBS and 100 IU/mL P/S [20]. Primary cortical neurons were prepared from the cortices of E15 C57BL6 mouse embryos. The cells were cultured at 37°C in Eagle’s basal medium (BME) (Gibco), supplemented with 10 % FBS, 100 IU/mL P/S, 2 mM L-glut, 25 mM KCl, and 10 μM cytosine β-d-arabinofuranoside (Sigma) [21].

### Expression Vectors and Transfections

The Gal4-TRAF and RelA fusion protein constructs were generated by cloning the individual TRAF and RelA cDNAs into the pBind vector (Promega). The TRAF constructs that were used for cotransfection with Gal4-tagged RelA were developed by cloning the individual TRAF cDNAs into the pCMV-Tag2B vector (Stratagene). Each of the cDNAs were amplified from the whole genome cDNA library (Promega) by polymerase chain reaction and sequenced. The TRAF2 N-terminal RING-finger deletion (ΔN-TRAF2) constructs were generated by removing a 261-bp DNA fragment corresponding to the N-terminal 87 amino acids of TRAF2 [22]. Cells were transfected using Lipofectamine 2000, according to the manufacturer’s instructions (Invitrogen).

### Protein Extraction

Whole cell extracts were prepared by lysing the cells in radioimmuno-precipitation assay buffer (50 mM Tris–HCl, pH 7.4, 150 mM NaCl, 1 % NP-40, 0.5 % sodium deoxycholate, 1 mM EDTA, 1 mM phenylmethyl-sulfonyl fluoride, 1 mM dithiothreitol (DTT), 1× complete protease inhibitor (Roche), and 1× phosphatase inhibitor cocktail I and II (Sigma)) [23]. To prepare cytoplasmic and nuclear extracts, the cells were first incubated for 30 min with buffer A (10 mM HEPES, pH 7.9; 1.5 mM MgCl_2_; 10 mM KCl; 0.5 mM DTT; 1× complete protease inhibitor; 1× phosphatase inhibitor cocktail I and II; 0.8 % NP-40) and centrifuged at 4600×*g* for 10 min. Following centrifugation, the cytoplasmic extracts were collected, and the isolated nuclei pellets were washed three times with phosphate-buffered saline (PBS), and then incubated for 30 min with buffer B (50 mM HEPES, pH 7.9; 0.4 M KCl; 0.75 mM MgCl_2_; 0.5 mM EDTA; 12.5 % glycerol; 0.5 mM DTT; 1× complete; 1× phosphatase inhibitor cocktail I and II). After 30 min, the samples were centrifuged at 18,300×*g* for 10 min, and the nuclear extracts were collected [21, 24]. All steps were carried out at 4 °C.

### Chromatin Binding Assays

For chromatin binding assays, the residual chromatin pellets following soluble nuclear protein extractions were washed with PBS, and then incubated in buffer B supplemented with deoxyribonuclease I (Sigma) for 2 h on ice. After 2 h, an equivalent volume of 2× high salt solution (300 mM NaCl; 2 % Triton X-100; 100 mM HEPES, pH 7.5; 20 % glycerol; 1× complete; 1× phosphatase inhibitor cocktail 1 and II) was added to the samples, followed by an additional hour incubation on ice. The samples were then centrifuged at 18,300×*g* for 15 min, and the chromatin extracts were collected [25]. All steps were carried out at 4 °C.

### Western Blotting

Protein extracts were resolved by sodium dodecyl sulfate-polyacrylamide gel electrophoresis, transferred to nitrocellulose membranes, and the membranes were blocked for 1 h in Tris-buffered saline-0.1 % Tween 20 (TBS-t) containing 5 % bovine serum albumin (BSA). Following overnight incubation with the primary antibodies in blocking solution at 4 °C, the membranes were washed three times and then probed with HRP-conjugated secondary antibodies for 1 h at room temperature (RT). The antibodies were visualized using an enhanced chemiluminescence kit (ECL; GE Healthcare). The primary antibodies that were used were anti-TRAF1 (sc-1831), anti-TRAF2 (sc-876), anti-TRAF3 (sc-949), anti-TRAF4 (sc-1921), anti-TRAF5 (sc-52967), anti-TRAF6 (sc-7221), anti-TRAF7 (sc-49545), anti-glucose transporter 3 (Glut3) (sc-30107), anti-heat shock protein 60 (HSP60) (sc-13115), anti-RNA polymerase II (RNA pol II) (sc-899) (Santa Cruz Biotechnologies), anti-RelA (#3036), anti-Lamin A/C (#2032), anti-histone deacetylase 1 (HDAC1) (#2062) (Cell Signaling Technologies), anti-histone H3 (#07-690) (Upstate Signaling Solutions), and anti-β-actin (A5441) (Sigma). After probing, the membranes were stripped in stripping buffer (62.5 mM Tris, pH 6.8; 0.4 % SDS; 100 mM 2-mercaptoethanol) for 40 min at 65 °C, washed in TBS-t, and then reblocked. As controls for both the loading and the relative purity of the cytoplasmic and nuclear extracts, Glut3 (26) and HSP60 (27) were used as cytoplasmic markers, while HDAC1 (28), Lamin A/C (29), and RNA Pol II were used as nuclear markers. Furthermore, additional validation, via blocking peptide, of the TRAF2 and TRAF antibodies was performed to ensure specificity as they were used also in the ChIP experiments (Supplemental Fig. 2).

### Electrophoretic Mobility Shift Assay (EMSA)

Oligonucleotide consensus sequences (Santa Cruz; Promega) were ^32^P-labeled with T4 polynucleotide kinase (Promega). Equal amounts of nuclear extracts were either untreated or preincubated with 200 ng of antibody to TRAF2 or TRAF3, or with 100-fold excess of unlabeled NF-kB or NFAT consensus elements. The samples were then incubated with the radiolabeled consensus sequence for 30 min at RT in DNA-binding buffer (10 mM Tris, pH 7.5; 50 mM NaCl; 1 mM MgCl_2_; 0.5 mM EDTA; 0.5 mM DTT; 4 % glycerol) supplemented with 125 μg/mL poly(dI-dC):poly(dI-dC) (Sigma). After 30 min, the samples were loaded onto a pre-electrophoresed 6 % polyacrylamide gel in 0.5× Tris-borate EDTA buffer, and separated at 150 V for ∼1.5 h. Gels were then fixed and dried, and autoradiograms were obtained.

### NF-kB Consensus Oligo Precipitation Assay

NF-kB consensus oligo-coated agarose beads were obtained from Santa Cruz Biotechnologies. Nuclear extracts were incubated with the beads overnight in DNA-binding buffer [20]. The following day, the beads were centrifuged at 12,000×*g* for 10 min at 4 °C. The samples were then washed three times with DNA-binding buffer and then incubated at 100 °C for 5 min in 2× SDS sample buffer (125 mM Tris–HCl, pH 6.8; 4 % SDS; 20 % glycerol; 0.2 M DTT; 0.02 mg/mL bromophenol blue). Finally, Western blots were performed on the fractionated samples, according to the protocol described above.

### Gal4 Luciferase Assays

The Gal4-TRAF and RelA fusion protein constructs were cotransfected into Neuro2a and HEK 293 cells along with either the 4x Gal4-minimal thymidine kinase (TK) promoter-driven luciferase plasmid (R. Evans, Salk Institute), the 5x Gal4 promoter-driven luciferase plasmid (Promega), or the NF-kB promoter-driven luciferase plasmid (Clontech). The Gal4-RelA fusion protein constructs were additionally transfected either with or without the flag-tagged TRAF protein constructs. At 24 h posttransfection, lysates were collected and luciferase assays were performed according to the Promega Dual-Luciferase Reporter Assay System Technical Manual (Promega).

### siRNA Transfection

TRAF2 siRNAs were procured from Dharmacon (Chicago, IL) as ON-TARGET plus SmartPools. Nontargeting siRNAs (siScr) were used as negative controls. siRNAs were reverse transfected into Neuro2a cells using Lipofectamine RNAimax (Invitrogen) as per the manufacturer’s protocol. Briefly, 50 nM siRNA was diluted in 0.5 mL of OPTIMEM reduced serum medium (Invitrogen) along with 5 μl of Lipofectamine RNAimax on each well of a six-well plate. Cells (1 × 105) in 8 mL of antibiotic-free media were added to the transfection mixture. Twenty-four hours later, the media was replaced with media with antibiotics. Efficiency of knockdown was assessed via quantitative PCR (Supplemental Fig. 3 B and C)

### Chromatin Immunoprecipitation (ChIP)

Neuro2a cells were fixed with 1 % formaldehyde (in PBS) for 10 min, washed in ice-cold PBS for 5 min, and then quenched with glycine stop buffer (125 mM glycine in PBS) for 5 min. Cells were subsequently washed in ice-cold PBS for 5 min. Cells were then scraped in PBS containing phenylmethylsulfonyl fluoride (PMSF), spun, and resuspended in lysis buffer (50 mM HEPES pH 8.0, 1 mM EDTA, 140 mM NaCl, 10 % glycerol, 0.5 % NP-40, and 0.25 % Triton X-100) to sit on ice for 30 min. Lysate was then homogenized with a type-B Dounce homogenizer using 15 strokes per sample. Nuclear fraction was then collected by pelleting and resuspended in 600 μL of sonication buffer (10 mM Tris pH 8.0, 1 mM EDTA, 140 mM NaCl, 1 % SDS, 0.1 % sodium deoxycholate, and 1 % Triton X-100). Cells were then sonicated using a Misonix S-4000 sonicator for 10 s at 20 % power. Ten microliters of the sheared DNA was aliquoted out and considered input DNA. For ChIP, 100 μL of sheared DNA was diluted 10-fold in buffer Y (16.7 mM Tris pH 8.1, 167 mM NaCl, 0.01 % SDS, 1.1 % Triton X-100, and 1.2 mM EDTA). Diluted sheared DNA was precleared with 2 μg of appropriate IgG antibody and salmon sperm-coated DNA agarose beads (Millipore) for 1 h at 4 °C. Beads were spun at 0.6 rcf for 1 min, and supernatant was moved to a fresh tube. Two micrograms of specific antibody was then used for overnight IP at 4 °C. Salmon sperm-coated DNA agarose beads were then added for 1.5-h incubation. Beads were spun at 0.6 rcf for 1 min. Beads were washed once in TSEI (0.1 % SDS, 1 % Triton X-100, 2 mM EDTA, 20 mM Tris pH 8.1, 150 mM NaCl), once in TSEII (0.1 % SDS, 1 % Triton X-100, 2 mM EDTA, 20 mM Tris pH 8.1, 500 mM NaCl), once in TSEIII (0.5 M LiCl, 1 % NP-40, 1 % sodium deoxycholate, 1 mM EDTA, 10 mM Tris pH 8.1, 150 mM NaCl) and then two times in TE (1 mM EDTA, 10 mM Tris pH 8.1) for 5 min each. DNA was then eluted twice using elution buffer (1 % SDS and 0.1 M NaHCO_3_) for 15 min each elution, 75 μL of buffer each time. Ten microliters of input DNA was diluted 1:10 with buffer Y. One hundred fifty microliter elutions and input DNA were then reverse cross-linked by adding NaCl to a final concentration 200 mM and incubating for 5 h or overnight at 65 °C. Proteins were removed by bringing elutions to a final concentration of 10 mM EDTA and 40 mM Tris pH 6.8 with 1.25 units of proteinase K for 1.5 h at 45 °C. DNA from elutions was then purified using a Qiagen pcr purification kit. DNA was used directly in qPCR reactions. Antibodies used were pRNA pol II (Abcam), H2Bub (clone 56, Millipore), TRAF2 (Santa-Cruz), TRAF3 (Santa Cruz), and p65 (Santa Cruz).

### Quantitative RT-PCR

RNA was isolated using the Qiagen RNAeasy kit as per the supplier’s protocol. cDNA synthesis (iScript cDNA Synthesis kit) and real-time PCR analysis (iQ SYBR Green Supermix) were performed using the protocol provided with the products (Bio-Rad, Hercules, CA). Then, DNA was quantified by real-time quantitative PCR using specific primers (for Icam-1: forward, 5′-CATCTCCTAAGATGACCTGC-3′, reverse, 5′-GTACACATTCCTGGTGACAT-3′; for TRAF2: forward, 5′-AGGCCAAGAATTCCTATGTG-3′, reverse, 5′-CTCACTGAGCTGCTATTCTT-3′; for p65: forward, 5′-CAGACCCAGGAGTGTTCACAGA-3′, reverse, 5′-ACATGGACACACCCTGGTTCA-3′; for Gapdh (used as control): forward, 5′-GGGTGTGAACCACGAGAAAT-3′, reverse, 5′-CCTTCCACAATGCCAAAGTT-3′). On the other hand, ChIP DNA was quantified by real-time quantitative PCR using Icam-1 promoter-specific primers (for p65 consensus/TSS: forward, 5′-CATTACTTCAGTTTGGAAATTCCTAGATC-3′, reverse, 5′-GGAACGAGGGCTTCGGTATT-3′; for Ctrl: forward, 5′-GCCTGTGAGTGTGCTCTCTG-3′, reverse, 5′-ACGACCTGAGAAGGGACCTG-3′). To note that the p65 consensus/TSS primers cover a 150-bp promoter sequence that includes the p65 consensus sequence and the transcriptional start site (TSS) of the Icam-1 gene. Real-time PCR were performed using the Roche Lightcycler II real-time PCR detection system (Roche). To avoid variations from different samples, the relative Icam-1 mRNA levels were normalized against GAPDH mRNA content of the same sample.

### Immunofluorescence and Confocal Microscopy

Subconfluent Neuro2a cells grow on cover slips. Cells were then rinsed with PBS buffer at pH 7.4 and fixed in ice-cold methanol for 10 min at −20 °C. After 45-min incubation in 10 % FBS to block nonspecific protein binding, the fixed cells were incubated with the following primary antibodies anti-TRAF2 (sc-876) or anti-TRAF3 (sc-949) diluted (1:300) in PBS containing 3 % low-fat milk at 37 °C for 2 h. After washing with PBS containing 3 % low-fat milk, the fixed cells were incubated with FITC-conjugated anti-IgG antibody (1:150, Sigma-Aldrich) diluted in PBS containing 3 % low-fat milk, at 37 °C for 1 h. After washing, the cover slips were mounted on slides using Prolong Gold antifade reagent (Invitrogen). Fluorescence microscopy was performed using a Zeiss LSM510/UV confocal microscope with a ×63 oil immersion objective. Immuno-labeled slides (*n* = 4–5 representative fields per slide) were sectioned optically at 0.5-μm intervals (one focal plane) through the cell monolayer to obtain the appropriate focal depth. Images were captured and collected using Axiovision 4.7 program.

### Statistical Analysis

Otherwise noted in the figure legend, data were presented as mean ± SE from three independent experiments. Statistical analysis was carried out with Student’s *t* test for PCR data and with one-way ANOVA followed by Tukey’s test for Luciferase data. *p* < 0.05 was considered as statistically significant difference.

## Results

### TRAF2 and TRAF3 Inducibly Translocate into the Nucleus of Neurons and Neuro2a cells

**Fig. 1:**
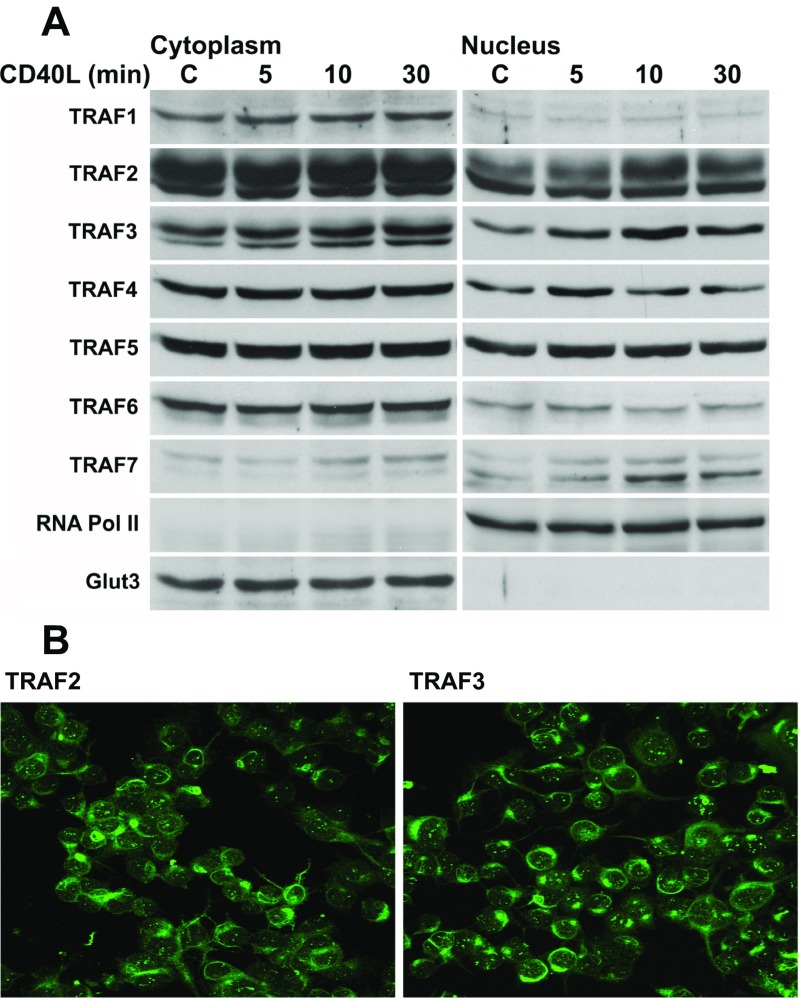
Analysis of the nuclear localization and translocation of TRAFs 1–7 in Neuro2a cells following CD40L stimulation. **a** Western blot analysis of the subcellular localization of TRAFs 1–7 in Neuro2a cells, as well as the effect of CD40L stimulation on the subcellular localization of TRAFs 1–7. Neuro2a cells were either untreated (control) or stimulated for 5, 10, or 30 min with mouse CD40L (100 ng/mL), cytoplasmic and nuclear extracts were then prepared, and a Western blot performed. As controls for both the loading and the relative purity of the cytoplasmic and nuclear extracts, Glut3 was used as a cytoplasmic marker, and RNA pol II was used as a nuclear marker. **b** Confocal images of TRAF2 and TRAF3 in Neuro2a cells showing subnuclear puncta

In order to determine whether the TRAF proteins can inducibly translocate into the nucleus, we first analyzed the subcompartmental localization of TRAFs 1–7 in Neuro2a cells following CD40L stimulation. It has previously been shown that both TRAFs 2 and 3 are degraded following several hours of CD40L stimulation [24]. For this reason, we analyzed the subcompartmental localization of the TRAFs following 5, 10, and 30 minutes of CD40L stimulation (Fig. 1a). We found that each of the TRAFs is variably present within the nucleus of Neuro2a cells. Also, in concordance with our Western blot data, TRAF2 and TRAF3 showed subnuclear localization in the form of puncta as well as a diffuse presence in the cytoplasm (Fig. 1b). Following CD40L stimulation, intranuclear protein levels of TRAFs 4, 5, and 6 appeared to increase maximally by 5 min, while TRAFs 2, 3, and 7 appear to increase maximally by 10 minutes, decreasing thereafter (Fig. 1a and Supplemental Fig. 1). TRAF1 was not induced to translocate into the nucleus of Neuro2a cells by 30 min of CD40L stimulation. Since TRAFs 2 and 3 had the most dramatic responses following CD40L stimulation, and because they are the most commonly studied TRAFs downstream of CD40 receptor signaling, we focused the following experiments on TRAFs 2 and 3.

**Fig. 2:**
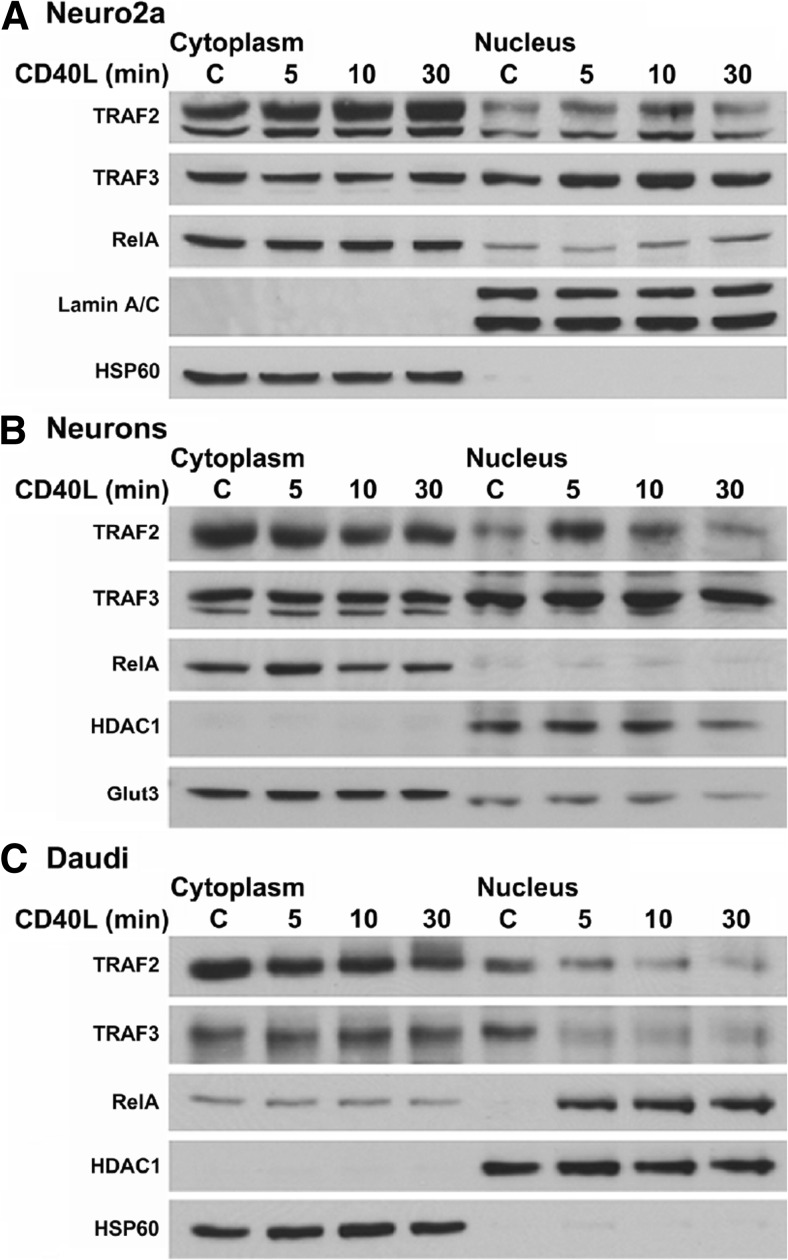
Analyses of the nuclear translocation of TRAF2, TRAF3, and RelA in Neuro2a cells, cortical neurons, and Daudi cells. **a** Western blot analysis of the effect of CD40L stimulation on the subcellular localization of TRAFs 2 and 3 in Neuro2a cells. Neuro2a cells were either untreated (control) or stimulated for 5, 10, or 30 min with mouse CD40L (100 ng/mL), cytoplasmic and nuclear extracts were then prepared, and a Western blot performed. As controls for both the loading and the relative purity of the cytoplasmic and nuclear extracts, HSP60 was used as a cytoplasmic marker, and Lamin A/C was used as a nuclear marker. **b** Western blot analysis of the effect of CD40L stimulation on the subcellular localization of TRAFs 2 and 3 in primary cortical neurons. E15 mouse cortical neurons were either untreated (control) or stimulated for 5, 10, or 30 minutes with mouse CD40L (100 ng/mL), cytoplasmic and nuclear extracts were then prepared, and a Western blot performed. As controls, Glut3 was used as a cytoplasmic marker and HDAC1 was used as a nuclear marker. **c** Western blot analysis of the effect of CD40L stimulation on the subcellular localization of TRAFs 2 and 3 in Daudi cells. Daudi cells were either untreated (control) or stimulated for 5, 10, or 30 min with human CD40L (100 ng/mL), cytoplasmic and nuclear extracts were then prepared, and a Western blot was performed. HSP60 and HDAC1 were used as controls. *C* control

We next investigated the translocation of TRAFs 2, 3, and p65/RelA in Neuro2a cells and mouse cortical neurons. We again found that TRAFs 2 and 3 translocate to the nucleus within 5 min of CD40L stimulation, peaking at 10 min, and decreasing to basal levels by 30 min of stimulation (Fig. 2a). This pattern of nuclear translocation differed from p65/RelA, which did not increase within the nucleus until 30 min of CD40L stimulation (Fig. 2a).

To determine whether our findings in Neuro2a cells were retained in primary cells, we repeated these experiments in mouse primary cortical neurons (Fig. 2b). We found that TRAF2 translocated into the nucleus by 5 min of stimulation, returning to basal levels by 30 minutes, while TRAF3 was present at constitutively high levels in the nucleus, and increased moderately following CD40L stimulation (Fig. 2b). Moreover, p65/RelA accumulation in the nucleus following CD40L stimulation was minimal (Fig. 2b). These data suggest that TRAF2, TRAF3 and p65/RelA translocate to the nucleus in CD40L stimulated Neuro2a cells and primary cortical neurons.

We next analyzed whether CD40L induced nuclear translocation of TRAFs 2 and 3 in a noncentral nervous system cell line, using pre-B lymphocytes (Daudi cells) as a model. Our results in Daudi cells differed significantly from neuronal cell types (Fig. 2c). Indeed, CD40L stimulation induced a strong reduction in the levels of both TRAFs 2 and 3 in the nucleus by 5 min, and these levels remained decreased at longer stimulation time points. In contrast, CD40L stimulation strongly induced the nuclear translocation of p65/RelA in these cells by 5 min, which remained increased by 30 min (Fig. 2c). These results demonstrate that the translocation of TRAFs 2 and 3 in response to CD40L stimulation functions in a cell-specific manner and may be tissue-specific.

### TRAF2 and TRAF3 Bind DNA

**Fig. 3:**
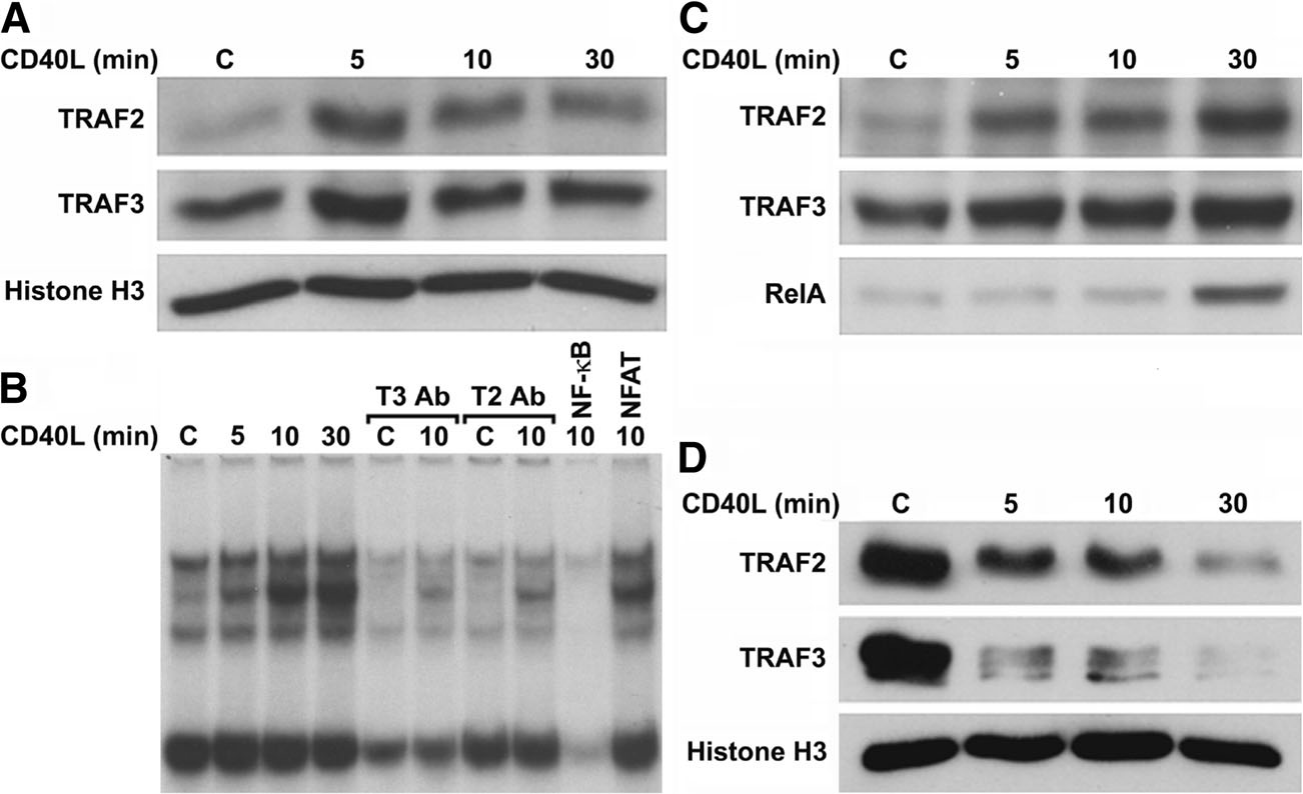
Inducible binding of TRAF2 and TRAF3 to chromatin and the NF-kB consensus element. **a** Western blot analysis of the binding of TRAFs 2 and 3 to chromatin in Neuro2a cells, following CD40L stimulation. Neuro2a cells were either untreated (control) or stimulated for 5, 10, or 30 min with mouse CD40L (100 ng/mL), chromatin extracts were then prepared, and a Western blot was performed. Histone H3 was used as a loading control and remained constant in all four conditions. **b** EMSA analysis of NF-kB DNA-binding activity in Neuro2a extracts following CD40L stimulation, and the effect of TRAF 2 or 3 antibody preincubation on such activity. Neuro2a cells were either untreated (control) or stimulated for 5, 10, or 30 min with mouse CD40L (100 ng/mL), nuclear extracts were then prepared, and an EMSA was performed. The extracts (5 μg) were then either untreated or preincubated with antibody to TRAF 2 or 3, or with 100-fold excess of unlabeled NF-kB or NFAT consensus elements, as controls for the specificity of the NF-kB DNA-binding complexes. Incubation with a 100-fold excess of unlabelled NF-kB promoter element greatly attenuated the DNA-binding of all four bands, while incubation with an excess of unlabeled NFAT element had no effect on any of the four bands, demonstrating that all four bands were specific. **c** Western blot analysis of the binding of TRAF2, TRAF3, and RelA to the NF-kB consensus element following NF-kB oligoprecipitation performed on CD40L-stimulated Neuro2a nuclear extracts. Neuro2a cells were either untreated (control) or stimulated for 5, 10, or 30 min with mouse CD40L (100 ng/mL), and nuclear extracts were prepared. The extracts (100 μg) were then incubated overnight with NF-kB-cross-linked agarose beads in DNA-binding buffer. The following day, the beads were precipitated and washed and a Western blot was performed. **d** Western blot analysis of the binding of TRAFs 2 and 3 to chromatin in Daudi cells, following CD40L stimulation. Daudi cells were either untreated (control) or stimulated for 5, 10, or 30 min with human CD40L (100 ng/mL), chromatin extracts were then prepared, and a Western blot was performed. Histone H3 was used as a loading control. *C* control, *T2* TRAF2, *T3* TRAF3, *Ab* antibody

We next evaluated whether, following nuclear translocation, TRAFs 2 and 3 were able to bind to chromatin, and more specifically to the NF-kB promoter element. For this purpose, we first prepared chromatin extracts and analyzed chromatin binding by Western blotting. We found that TRAF2 was minimally bound to chromatin, while there appeared to be more TRAF3 bound, in unstimulated Neuro2a cells (Fig. 3a). Surprisingly, CD40L enhanced chromatin binding of both TRAFs 2 and 3 by 5 min of stimulation, reducing thereafter. These results show for the first time that TRAFs 2 and 3 constitutively and inducibly bind chromatin, suggesting that the TRAFs may be direct regulators of transcriptional activity.

We next evaluated the possible binding of TRAFs 2 and 3 to the NF-kB consensus element by EMSA. NF-kB DNA-binding activity was constitutively present in unstimulated Neuro2a nuclear extracts, in the form of four differentially sized bands (Fig. 3b). The intensity of the upper three bands progressively increased following 5, 10, and 30 min of CD40L stimulation. However, preincubation of control and 10-min nuclear extract samples with antibodies to either TRAFs 2 or 3 greatly attenuated DNA-binding activity. These results suggest that TRAFs 2 and 3 constitutively and inducibly bind to the NF-kB promoter element in a time-dependent manner following CD40L stimulation. Furthermore, both proteins are essential components of their respective DNA-binding complexes because blocking their interactions by antibody preincubation attenuated DNA-binding activity.

To further demonstrate inducible binding of TRAFs 2 and 3 to the NF-kB promoter element, we conducted an NF-kB promoter element oligoprecipitation assay. Similar to the EMSA experiment, both TRAFs 2 and 3 bound to the NF-kB consensus element in unstimulated extracts, with TRAF3 bound to a greater degree than TRAF2 (Fig. 3c). CD40L stimulation induced the binding of both TRAFs 2 and 3 to the NF-kB consensus element in a time-dependent manner, with maximum binding after 30 min of stimulation. In addition, p65/RelA inducibly bound to the NF-kB consensus element following 30 min of CD40L stimulation, in direct correlation to the increased levels of p65RelA in CD40L-stimulated nuclear extracts (Fig. 2b). Collectively, these results support our EMSA analyses and show that TRAFs 2 and 3 both constitutively and inducibly bind to the NF-kB promoter element and form part of a transcriptional complex containing TRAFs 2 and 3 along with p65/RelA.

Considering the differences between the nuclear translocation of TRAFs 2 and 3 between Neuro2a and Daudi cells, we next analyzed the chromatin binding of these proteins in Daudi cells. The levels of TRAFs 2 and 3 binding to chromatin correlated with their nuclear levels (compare Fig. 3d to Fig. 2c). Particularly, both TRAFs 2 and 3 were constitutively bound to chromatin at high levels, and this binding greatly diminished following CD40L stimulation. These results further show that the translocation and chromatin binding of TRAFs 2 and 3 may function in a cell-specific manner, but also suggest that the TRAFs may have a role in regulating transcriptional activity in Daudi cells as well.

### TRAFs Differentially Function as Coactivators and/or Corepressors of Transcription

**Fig. 4:**
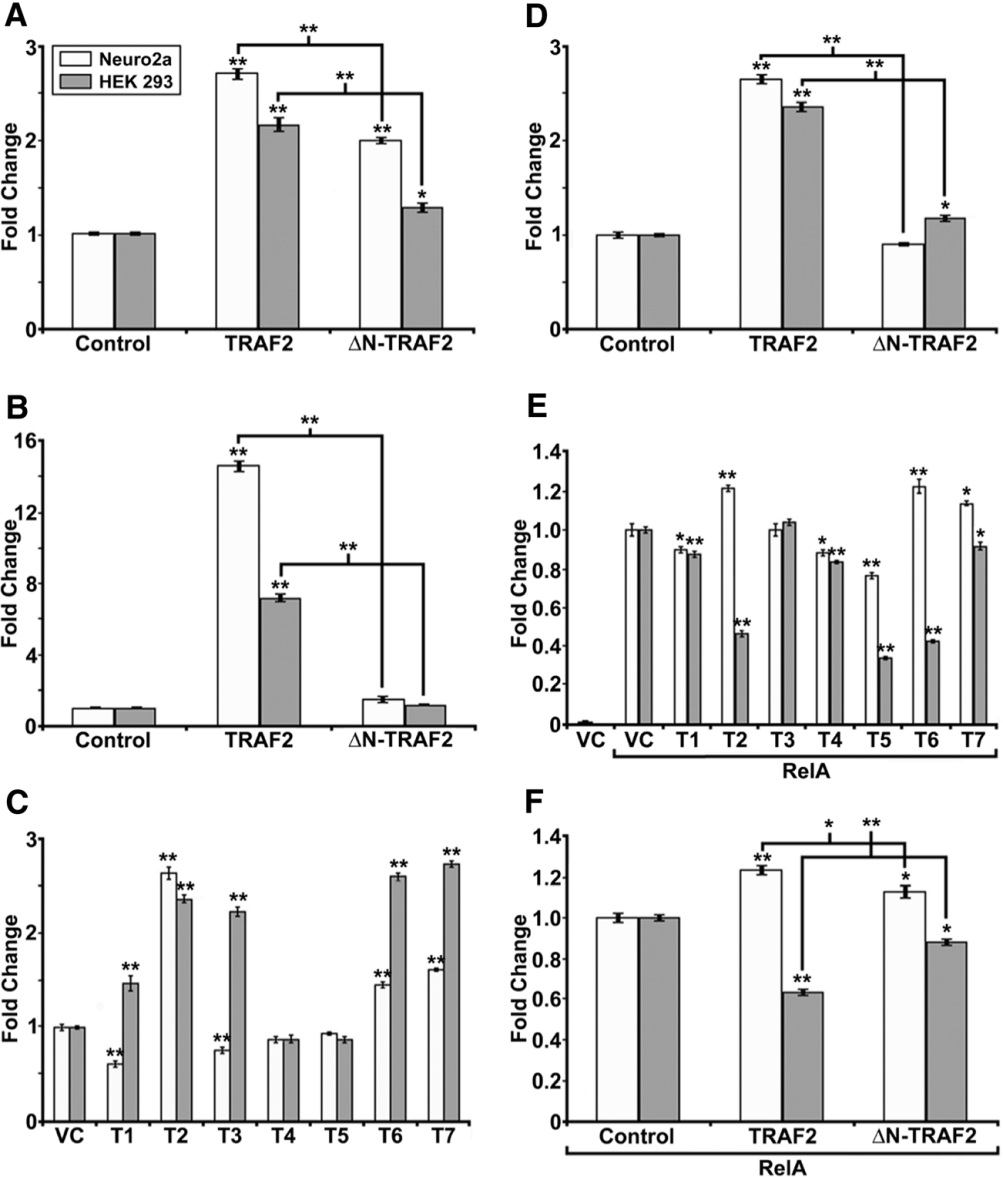
Transcriptional regulatory potentials of TRAFs 1–7. **a** NF-kB dual-luciferase assay performed on Neuro2a (*light gray*) and HEK 293 (*dark gray*) cell extracts , with the overexpression the pBind vector, pBind-TRAF2, or pBind-ΔN-TRAF2. Neuro2a and HEK 293 cells were transfected either with the control pBind vector or with pBind-TRAF2 or pBind-ΔN-TRAF2 (1 μg each). After 24 h, the cells were lysed and dual-luciferase assays were performed. **b** 5x Gal4 promoter-driven dual-luciferase assay performed on Neuro2a and HEK 293 cell extracts, with the overexpression of the pBind vector, pBind-TRAF2, or pBind-ΔN-TRAF2. **c** 4x Gal4-minimal TK promoter-driven dual-luciferase assay performed on Neuro2a and HEK 293 cell extracts, with the overexpression of the pBind vector or pBind-TRAF1-7. **d** 4x Gal4-minimal TK promoter-driven dual-luciferase assay performed on Neuro2a and HEK 293 cell extracts, with the overexpression of the pBind vector, pBind-TRAF2, or pBind-ΔN-TRAF2. **e** 5x Gal4 promoter-driven dual-luciferase assay performed on Neuro2a and HEK 293 extracts, with the overexpression of the pBind vector or pBind-RelA, in turn with either the pCMV-Tag2C vector or pCMV-Tag2B-TRAF1-7. **f** 5x Gal4 promoter-driven dual-luciferase assay performed on Neuro2a and HEK 293 cell extracts overexpressed with pBind RelA, in turn coexpressed with the pCMV-Tag2B vector, pCMV-Tag2B-TRAF2 or pCMV-Tag2B-ΔN-TRAF2. Each of these experiments was repeated at least three times, and the data represent the mean of at least four independent sample readings from one representative experiment ± the SEM, normalized to the pBind Renilla luciferase control readings. One-way ANOVA was performed, followed by Tukey’s test; *≤0.05; **≤0.001. *VC* vector control, *T1* TRAF1, *T2* TRAF2, *T3* TRAF3, *T4* TRAF4, *T5* TRAF5, *T6* TRAF6, *T7* TRAF7

Previous studies have indicated that overexpression of TRAFs 2 and 6 can induce NF-kB transcriptional activity [16, 22, 26, 27]. Since we demonstrated that TRAFs 2 and 3 bind to chromatin, we next analyzed whether the TRAF proteins could activate transcription using a luciferase assay reporter system (Fig. 4). As previously shown, we demonstrated that overexpressing TRAF2 induces NF-kB transcriptional activity in Neuro2a (2.7-fold) and HEK 293 cells (2.2-fold) (Fig. 4a) [14]. In consideration of the previous findings that TRAF2 functions as an E3-ubiquitin ligase via the RING finger domain, and that deletion of the RING finger can attenuate its ability to induce transcriptional activity [9, 10, 22], we repeated the overexpression experiment using a deletion construct lacking the RING finger domain, ΔN-TRAF2. We found that deletion of the RING finger domain reduces TRAF2 induced NF-kB transcriptional activity.

In order to determine the direct transactivation potential of TRAF2, we next analyzed the transcriptional activity induced by TRAF2 coexpressed with a 5x Gal4 promoter-driven luciferase reporter plasmid (Fig. 4b). As a result, overexpression of TRAF2 induced a 14.6-fold increase in luciferase activity in Neuro2a cells, and a 7.2-fold increase in HEK 293 cells. In this experiment, deletion of the RING finger domain completely abolished the transactivation potential of TRAF2 in both Neuro2a and HEK 293 cells. These results suggest that TRAF2 can function directly as a transcription factor.

Based on the finding that TRAF2 functions as a direct transcriptional activator, we further explored the possibility that the TRAFs could function as coactivators or corepressors. In order to determine the coactivation/corepression potential of the TRAFs, we coexpressed Gal4-tagged isoforms of TRAF1-7 along with a 4x Gal4-minimal TK promoter-driven luciferase reporter plasmid. This reporter plasmid maintains a basal level of transcriptional activity, allowing for the analysis of transcriptional repression [28]. We found that each of the TRAFs functioned as a coactivator or corepressor for the minimal TK promoter activity in both Neuro2a and HEK 293 cells, with the exception of TRAFs 4 and 5, which had no effect in either cell type (Fig. 4c). In Neuro2a cells, TRAFs 2, 6, and 7 were found to coactivate the minimal TK promoter activity, while TRAFs 1 and 3 were found to corepress it. In HEK 293 cells, TRAFs 1, 2, 3, 6, and 7 all had a coactivator function. Surprisingly, while the majority of the TRAF proteins maintained similar coactivation or corepression patterns between both Neuro2a and HEK 293 cells, TRAFs 1 and 3 had very different functions between the two cell types, again suggesting that particular TRAF proteins may have cell-specific functions. In order to determine the role of the RING finger domain in the coactivation activity of TRAF2 in both Neuro2a and HEK 293 cells, we next repeated the experiment with the ΔN-TRAF2 construct, and found that deletion of the RING finger domain abolished the coactivation potential of TRAF2 in both cell types (Fig. 4d).

Since the TRAFs can function as coactivators and corepressors, we next evaluated whether coexpression of the TRAFs could have an effect on coactivating or corepressing Gal4-tagged RelA-induced transcriptional activity (Fig. 4e). In Neuro2a cells, TRAFs 2, 6, and 7 enhanced, while TRAFs 1, 4, and 5 repressed RelA-induced, transcriptional activity. In HEK 293 cells, TRAFs 1, 2, 4, 5, 6, and 7 all repressed RelA-induced transcriptional activity. In both cell types, TRAF3 had no effect on RelA-induced transcriptional activity. Additionally, deletion of the RING finger domain from TRAF2 attenuated the coactivation or corepression activity of TRAF2 for RelA-induced transcriptional activity in Neuro2a and HEK 293 cells, respectively (Fig. 4f). Taken together, these results show that the TRAF proteins can function as coactivators or corepressors of a known transcription factor, RelA, dependent upon the cell type, and that their coactivation or corepression potential may be due in part to the E3 ubiquitin-ligase activity of their RING finger domains.

### CD40L Induces a Transcriptional Complex Consisting of pRNA PolII, TRAF2, TRAF3, and p65 in Neuro2a Cells

**Fig. 5:**
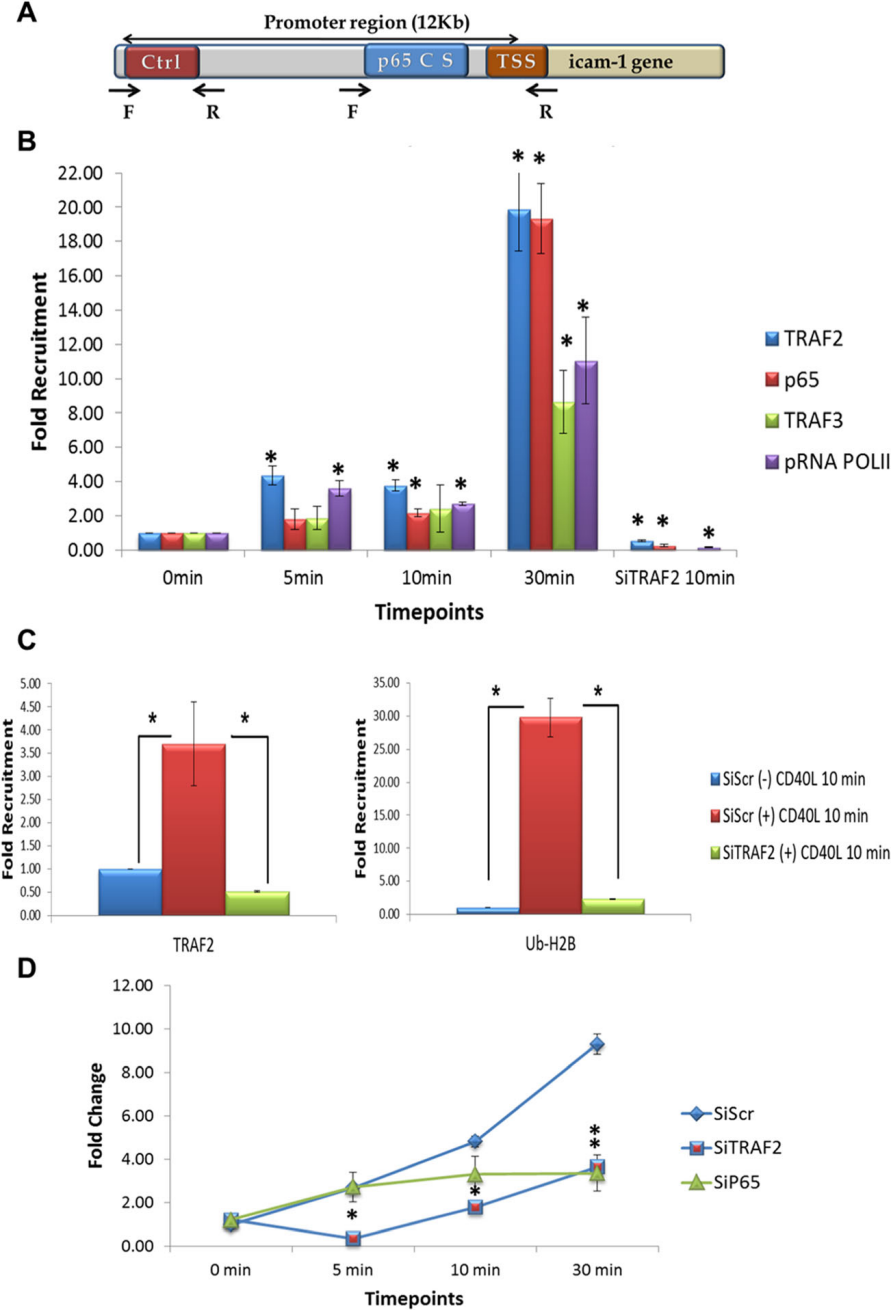
TRAF2 regulates mouse Icam-1 gene expression upon CD40L stimulation in Neuro2a cells. **a** Schematic diagram of mouse Icam-1 gene promoter. **b** Time course of TRAF2, pRNAPolII, and p65 recruitment to mouse icam-1 promoter upon CD40L treatment in Neuro2a cells. qPCR analysis of TRAF2, p65, TRAF3, and pRNAPolII chromatin immunoprecipitation (ChIP) after 5, 10, and 30 min of CD40L (100 ng/mL) treatment. ChIP qPCR analysis shows significant increase in TRAF2 and pRNAPolII recruitment to p65 consensus sequence (p65 C S) starting 5 min after CD40L treatment. At 10 min, p65 joined TRAF2 and pRNAPolII at the promoter. Following 30 min, TRAF3 joined the complex and maximal levels of TRAF2, pRNAPolII and p65 occurred at this time. Knockdown of TRAF2 by SiRNA (SiTRAF2) abrogated p65, and pRNAPolII to the complex on the Icam-1 promoter. The TRAF3 recruitment to the promoter was not investigated since it did not show any changes in levels at the tested 10-min time point. Nonspecific IgG was used as an internal control while primers flanking an irrelevant control sequence were used as external controls. **c** Monoubiquitination of H2B and TRAF2 recruitment to the Icam-1 gene promoter. *Left panel*: qPCR analysis of TRAF2 ChIP after 10 min of CD40L (100 ng/mL) treatment show 3.7-fold increase in TRAF2 recruitment to p65 consensus sequence (p65 C S) as compared to nontreated cells. This increase is abrogated when TRAF2 was knocked down using SiRNA. *Right panel*: qPCR analysis of H2B-Ub (Lys 120) ChIP after 10 min of CD40L (100 ng/mL) treatment show 29.8-fold increase in H2B monoubiquitination at the p65 consensus sequence (p65 C S) as compared to nontreated cells. This increase is abrogated when TRAF2 was knocked down using SiRNA. Nonspecific IgG was used as an internal control while primers flanking an irrelevant control sequence were used as external controls. **d** Mouse Icam-1 mRNA expression levels upon CD40L treatment in Neuro2a cells. qPCR analysis of Icam-1 mRNA expression in Neuro2a cells treated with Scrambled, TRAF2, and p65 SiRNA at 0, 5, 10, and 30 min after CD40L (100 ng/mL) treatment. TRAF2 knockdown shows dramatic decrease in Icam-1 mRNA expression starting 5 min while p65 knockdown significantly affects Icam-1 expression at the 30-min time point. *TSS* transcriptional start site. *Ctrl* irrelevant control sequence, *F* and *R* forward and reverse primers, respectively. *p < 0.05

Based upon the data described above, we wanted to determine if CD40L could stimulate TRAF2 and or TRAF3 binding to an NF-kB-dependent promoter, in vivo. To investigate this, we performed ChIP-qPCR analysis of the Icam-1 promoter in Neuro2A cells. Expression of Icam-1 was shown to be rapidly induced upon CD40L activation [3, 4, 29, 30]. A diagram of the Icam-1 promoter is shown if Fig. 5a.

In these experiments, Neuro2A cells were treated with media or CD40L for 5–30 min and ChIP-qPCR analysis was performed. Our data demonstrates that TRAF2 and phospho-RNA Polymerase II (pRNA PoIII) are recruited to the NF-kB consensus element within 5 min of CD40L stimulation, prior to either p65/RELA or TRAF3 promoter occupancy (Fig. 5b). Significant binding of p65/RelA and TRAF3 is further observed at 10 and 30 min poststimulation, respectively (Fig. 5b). Maximal binding of TRAF2, pRNA PoIII, p65/RelA, and TRAF3 occurred at 30 min of CD40L stimulation.

### TRAF2 Containing Complexes Are Critical for H2B-Ubiquitination at the Icam-1 Promoter and Regulate Icam-1 Gene Expression

Since TRAF2 recruitment to the Icam-1 promoter was an initial event preceding recruitment of other complex members, we investigated TRAF2’s effects on histone H2B Lys120 mono-ubiquitination (H2Bub), a posttranslational modification associated with the facilitation of transcription initiation and elongation [31]. ChIP experiments revealed a significant increase in H2Bub levels upon 10 min of CD40L stimulation in Neuro2A cells (Fig. 5c). The increased occupancy by H2Bub was specific for the NF-kB consensus site, as compared to the control region of the Icam-1 gene promoter. On the other hand, an early transcription repressive epigenetic modification as in ubiquitination of histone H2A was probed and no significant difference in H2Aub occupancy was seen after 10 min of CD40L treatment (Supplemental Fig. 3A). Next, we show that siRNA knockdown of TRAF2 significantly inhibited recruitment of TRAF2, p65, pRNAPolII to the promoter and H2Bub and (Fig. 5b, c). Finally, we demonstrate that knocking down TRAF2 and p65 significantly inhibits Icam-1 gene transcription following CD40L stimulation. Interestingly, inhibition of TRAF2 significantly repressed Icam-1 mRNA generation as early as 5 min and for up to 30 min whereas inhibiting p65 attenuated Icam-1 expression only at 30 min of stimulation (Fig. 5d). As a control, we demonstrate knockdown of TRAF2 and p65 mRNAs by their specific siRNAs (Supplemental Fig. 3B and C).

## Discussion

In this study, we demonstrated, for the first time, that the TRAFs are variably present within the nucleus of Neuro2a cells and primary cortical neurons and that TRAFs 2 and 3 rapidly translocate into the nucleus following treatment with CD40L. Our in vitro studies have shown that both TRAFs 2 and 3 bind to chromatin and the NF-kB promoter element and can function as coactivators or corepressors of transcriptional activity. Additionally, TRAF2 can directly induce transcriptional activity, albeit weakly in comparison to typical transcription factors. We find these results extremely interesting for a number of reasons, including how rapidly the TRAFs translocate into the nucleus and associate with chromatin, that they have the capacity to function as transcriptional regulators and that they may have different functions and responses within different cell types or even with different target transcription factors within the same cell. In support of our in vitro data, we determined using ChIP-qPCR analysis that TRAFs 2 and 3 form part of an active transcriptional complex at the Icam-1 promoter and that TRAF2 is required for p65 and pRNAPolII binding. We further demonstrate that a complex containing TRAF2 ubiquitinates H2b. These findings demonstrate a novel function for the TRAFs in the nucleus, as the TRAFs have been previously considered to function exclusively within the cytoplasm either as adaptor proteins and or as E3-ubiquitin ligases.

Interestingly, an ever increasing number of ubiquitin ligases have been found to function within the nucleus [32]. These proteins can function as coactivators or corepressors, regulating transcription factors either by enhancing their activation or inducing their degradation or processing [33, 34]. In this case, it is very possible that the TRAFs may function to regulate transcriptional activity in a similar manner, as their rapid regulation of transcriptional activation could have a wide-ranging, direct regulatory effect that would explain the number of pathways and functions that the various TRAFs have been found to regulate. Additionally, since both TRAFs 2 and 6 have been found to catalyze Lys-63-linked polyubiquitination [35, 36], this suggests that the TRAFs may also directly activate proteins, rather than solely signal proteins for degradation or proteasomal processing. However, in several cases, the transcriptional regulatory capacity of TRAF2 was not entirely abolished by deletion of the RING finger, and TRAF1 has the capacity to function as a corepressor without having a RING finger domain. Therefore, this suggests that additional domains must have transcriptional regulatory capacity and will require additional research in order to parse out such functions.

Considering that several of the TRAFs were found to have different transcriptional activities between Neuro2a and HEK 293 cells, it is possible that the individual TRAFs do not have a specific coactivator or corepressor potential. In fact, as some of the TRAFs have a coactivator activity in one cell type and a corepressor activity in another cell type (namely TRAFs 1 and 3 as coactivators in HEK 293 cells and corepressors in Neuro2a cells), it is possible that the functions of the various TRAFs may depend on the intracellular milieu, as defined by the proteins and cofactors that are individually present within the different cell types, the stimulus that activates them, and the specific target protein(s) that they are regulating. Indeed, TRAF2 functions as a coactivator for the minimal TK promoter activity in HEK 293 cells, but as a corepressor for RelA-induced activity in the same cell type. Additionally, neither TRAF4 nor TRAF5 function as a coactivator or corepressor of the minimal TK promoter activity in either Neuro2a or HEK 293 cells, while both function as corepressors for RelA-induced transcriptional activity in both cell types. Finally, a clearer example of this is the finding that TRAF3 functions as corepressor of the minimal TK promoter activity in Neuro2a cells, and as a coactivator in HEK 293 cells, without having any effect on RelA-induced transcriptional activity in either cell type.

Another interesting finding is that TRAFs 2 and 3 translocate into the nucleus of Neuro2a cells and primary cortical neurons following CD40L stimulation, while in Daudi cells, they rapidly exit the nucleus or are quickly degraded. This suggests further differences in the mechanisms of TRAF signaling between cell types, in addition to the differences in transcriptional regulatory potentials. We cannot exclude, however, that in Daudi cells, other TRAFs may translocate into the nucleus following CD40L stimulation, or that stimulation with cytokines other than CD40L may have other effects. Indeed, nuclear extracts from unstimulated Daudi cells had high constitutive levels of both TRAFs 2 and 3, and both were constitutively bound to chromatin, suggesting that both proteins may have a function within the nucleus of Daudi cells, as well.

**Fig. 6:**
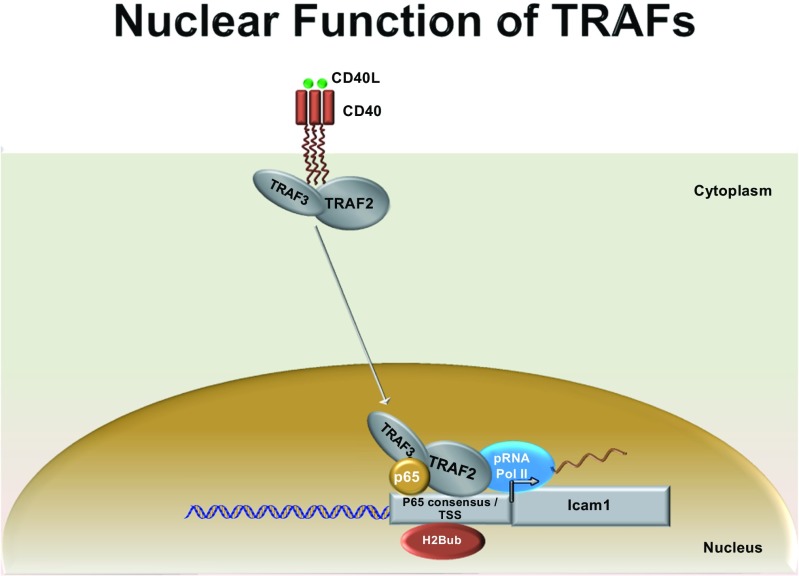
Model schema describing novel transcriptional capabilities of TRAFs 2 and 3 in neurons. TRAFs 2/3, p65, and pRNAPolII form part of a transcriptional complex on Icam-1 gene promoter upon CD40L stimulation. TRAF2 recruitment to the nucleus is critical for the monoubiquitination of H2B, a transcription permissive epigenetic modification

Overall, our data show for the first time that the TRAF proteins can translocate into the nucleus, bind to both chromatin and the NF-kB consensus element. Additionally, we show that TRAF2 and TRAF3 are components of DNA-binding complexes containing p65 and pRNA PolII, and that this complex epigenetically modifies H2b at the Icam-1 promoter (Fig. 6). This is very intriguing because it suggests that inducible transcriptional complexes containing different TRAF proteins form at the Icam-1, and possibly other promoters in a temporal specific pattern. Our data also demonstrates that TRAF2 participates in the orchestration of histone H2 ubiquitination, which contributes to the selective regulation of NF-kB-dependent genes. Finally, our observation of TRAF3 binding to chromatin at 5 min post-CD40L stimulation (Fig. 4a), but not at the Icam-1 promoter at this time point (Fig. 5b) suggests that the temporal occupancy and the composition of the TRAF transcriptional complexes is context dependent.

Overall, these findings are important given the diverse functions that continue to be attributed to the wide range of receptor families (e.g., TNFR, TLR, and NLR) that utilize the TRAFs to orchestrate signaling. Future research into the genes that are regulated by the specific TRAF complexes will further elucidate how the TRAFs regulate signaling, as well as whether dysfunctions in TRAF signaling may be associated with known disorders. If specific TRAF complexes are found to regulate specific genes, then pharmacological targeting of individual TRAF complexes may allow for the highly specific inhibition of signaling events downstream of these receptors, without compromising overall receptor signaling, transcription factor pathways, or cellular systems.

## Electronic supplementary material

Below is the link to the electronic supplementary material.

Supplemental Figure 1 Quantification of the nuclear translocation of TRAF2 and TRAF3 in Neuro2a cells following CD40L-stimulation. The data represent the means of three independent experiments conducted in Fig. 2a +/- the SEM, quantified and normalized to Lamin A/C. One way ANOVA was performed, followed by Tukey’s test. * p ≤ 0.05. (PPTX 154 kb)

Supplemental Figure 2 Verification of the specificity of the TRAF antibodies. A Western blot was performed with unstimulated Neuro2a whole cell extract (30 μg). The membrane was probed with an antibody to TRAF2 or TRAF3, either with or without preadsorption to their respective blocking peptide. Preadsorption was performed by incubating the antibodies at 4^o^C overnight with 10-fold excess blocking peptide. β-Actin was used as a control. Ab = antibody. (PPTX 403 kb)

Supplemental Figure 3 **A)** monoubiquitination of H2A upon CD40L stimulation. qPCR analysis of H2A-Ub (Lys 120) ChIP after 10 minutes of CD40L (100 ng/ml) treatment show insignificant H2A monoubiquitination at the p65 consensus sequence as compared to non treated cells. Non-specific IgG was used as an internal control while primers flanking an irrelevant control sequence were used as external controls. **B)** and **C)** Efficiency of TRAF2 and p65 knockdown in Neuro2a cells. qPCR analysis of TRAF2 **(B)** and p65 **(C)** mRNA expression show 95% and 93% knockdown efficiency respectively in Neuro2a cells as compared to Scrambled SiRNa (SiScr). (PPTX 117 kb)

## Abbreviations

TRAF: Tumor necrosis factor receptor-associated factor
TNFR: TNF receptor
NLS: Nuclear localization sequence
NF-kB: Nuclear factor-kappa B
MEM: Minimum essential medium
DMEM: Dulbecco’s modified Eagle’s medium
FBS: Fetal bovine serum
P/S: Penicillin/streptomycin
l-Glut: l-Glutamine
BME: Eagle’s basal medium
ΔN-TRAF2: N-terminal RING-finger deletion mutant TRAF2
DTT: Dithiothreitol
PBS: Phosphate-buffered saline
TBS-t: Tris-buffered saline-0.1 % Tween 20
BSA: Bovine serum albumin
RT: Room temperature
ECL: Enhanced chemiluminescence
Glut3: Glucose transporter 3
HSP60: Heat shock protein 60
RNA pol II: RNA polymerase II
HDAC1: Histone deacetylase 1
EMSA: Electrophoretic mobility shift assay
TK: Thymidine kinase

## References

[1] Hacker H, Tseng PH, Karin M (2011). Expanding TRAF function: TRAF3 as a tri-faced immune regulator. Nat Rev Immunol.

[2] Xie P (2013). TRAF molecules in cell signaling and in human diseases. J Mol Signal.

[3] Barbe-Tuana FM (2006). CD40-CD40 ligand interaction activates proinflammatory pathways in pancreatic islets. Diabetes.

[4] Henriquez NV (1999). Differential responses to CD40 ligation among Burkitt lymphoma lines that are uniformly responsive to Epstein-Barr virus latent membrane protein 1. J Immunol.

[5] Inoue J (2000). Tumor necrosis factor receptor-associated factor (TRAF) family: adapter proteins that mediate cytokine signaling. Exp Cell Res.

[6] Ostuni R, Zanoni I, Granucci F (2010). Deciphering the complexity of Toll-like receptor signaling. Cell Mol Life Sci.

[7] Wajant H, Henkler F, Scheurich P (2001). The TNF-receptor-associated factor family: scaffold molecules for cytokine receptors, kinases and their regulators. Cell Signal.

[8] Wajant H, Scheurich P (2001). Tumor necrosis factor receptor-associated factor (TRAF) 2 and its role in TNF signaling. Int J Biochem Cell Biol.

[9] Habelhah H (2010). Emerging complexity of protein ubiquitination in the NF-kappaB pathway. Genes Cancer.

[10] Habelhah H (2004). Ubiquitination and translocation of TRAF2 is required for activation of JNK but not of p38 or NF-kappaB. Embo J.

[11] Napetschnig J, Wu H (2013). Molecular basis of NF-kappaB signaling. Annu Rev Biophys.

[12] Xu LG, Li LY, Shu HB (2004). TRAF7 potentiates MEKK3-induced AP1 and CHOP activation and induces apoptosis. J Biol Chem.

[13] Chen ZJ (2012). Ubiquitination in signaling to and activation of IKK. Immunol Rev.

[14] Cheng G (1995). Involvement of CRAF1, a relative of TRAF, in CD40 signaling. Science.

[15] Hu HM (1994). A novel RING finger protein interacts with the cytoplasmic domain of CD40. J Biol Chem.

[16] Rothe M (1994). A novel family of putative signal transducers associated with the cytoplasmic domain of the 75 kDa tumor necrosis factor receptor. Cell.

[17] Sato T, Irie S, Reed JC (1995). A novel member of the TRAF family of putative signal transducing proteins binds to the cytosolic domain of CD40. FEBS Lett.

[18] Kedinger V, Rio MC (2007). TRAF4, the unique family member. Adv Exp Med Biol.

[19] Krajewski S (1997). Immunohistochemical analysis of in vivo patterns of TRAF-3 expression, a member of the TNF receptor-associated factor family. J Immunol.

[20] Morita Y (2005). TRAF7 sequesters c-Myb to the cytoplasm by stimulating its sumoylation. Mol Biol Cell.

[21] Glauner H (2002). Intracellular localization and transcriptional regulation of tumor necrosis factor (TNF) receptor-associated factor 4 (TRAF4). Eur J Biochem.

[22] Min W (1998). The N-terminal domains target TNF receptor-associated factor-2 to the nucleus and display transcriptional regulatory activity. J Immunol.

[23] Gamper C (2000). TRAF-3 interacts with p62 nucleoporin, a component of the nuclear pore central plug that binds classical NLS-containing import complexes. Mol Immunol.

[24] Moore CR, Bishop GA (2005). Differential regulation of CD40-mediated TNF receptor-associated factor degradation in B lymphocytes. J Immunol.

[25] Tan J (2002). CD40 is expressed and functional on neuronal cells. Embo J.

[26] Ishida T (1996). Identification of TRAF6, a novel tumor necrosis factor receptor-associated factor protein that mediates signaling from an amino-terminal domain of the CD40 cytoplasmic region. J Biol Chem.

[27] Rothe M (1995). TRAF2-mediated activation of NF-kappa B by TNF receptor 2 and CD40. Science.

[28] Chen JD, Evans RM (1995). A transcriptional co-repressor that interacts with nuclear hormone receptors. Nature.

[29] Saito M (2007). CD40-CD40 ligand signal induces the intercellular adhesion molecule-1 expression through nuclear factor-kappa B p50 in cultured salivary gland epithelial cells from patients with Sjogren's syndrome. Mod Rheumatol.

[30] von Leoprechting A (1999). Stimulation of CD40 on immunogenic human malignant melanomas augments their cytotoxic T lymphocyte-mediated lysis and induces apoptosis. Cancer Res.

[31] Hammond-Martel I, Yu H, Affar el B (2012). Roles of ubiquitin signaling in transcription regulation. Cell Signal.

[32] Natoli G, Chiocca S (2008). Nuclear ubiquitin ligases, NF-kappaB degradation, and the control of inflammation. Sci Signal.

[33] Daniels MJ, Marson A, Venkitaraman AR (2004). PML bodies control the nuclear dynamics and function of the CHFR mitotic checkpoint protein. Nat Struct Mol Biol.

[34] Tanaka T, Grusby MJ, Kaisho T (2007). PDLIM2-mediated termination of transcription factor NF-kappaB activation by intranuclear sequestration and degradation of the p65 subunit. Nat Immunol.

[35] Lamothe B (2007). Site-specific Lys-63-linked tumor necrosis factor receptor-associated factor 6 auto-ubiquitination is a critical determinant of I kappa B kinase activation. J Biol Chem.

[36] Pineda G, Ea CK, Chen ZJ (2007). Ubiquitination and TRAF signaling. Adv Exp Med Biol.

