# Supplementary figures and images for: Tumor Necrosis Factor Receptor Associated Factors (TRAFs) 2 and 3 Form a Transcriptional Complex with Phosho-RNA Polymerase II and p65 in CD40 Ligand Activated Neuro2a Cells
Source: Mol Neurobiol. 2016 Feb 3;54(2):1301–13. doi: 10.1007/s12035-016-9742-4 (PMC5310569; doi:10.1007/s12035-016-9742-4)

## Slide 1
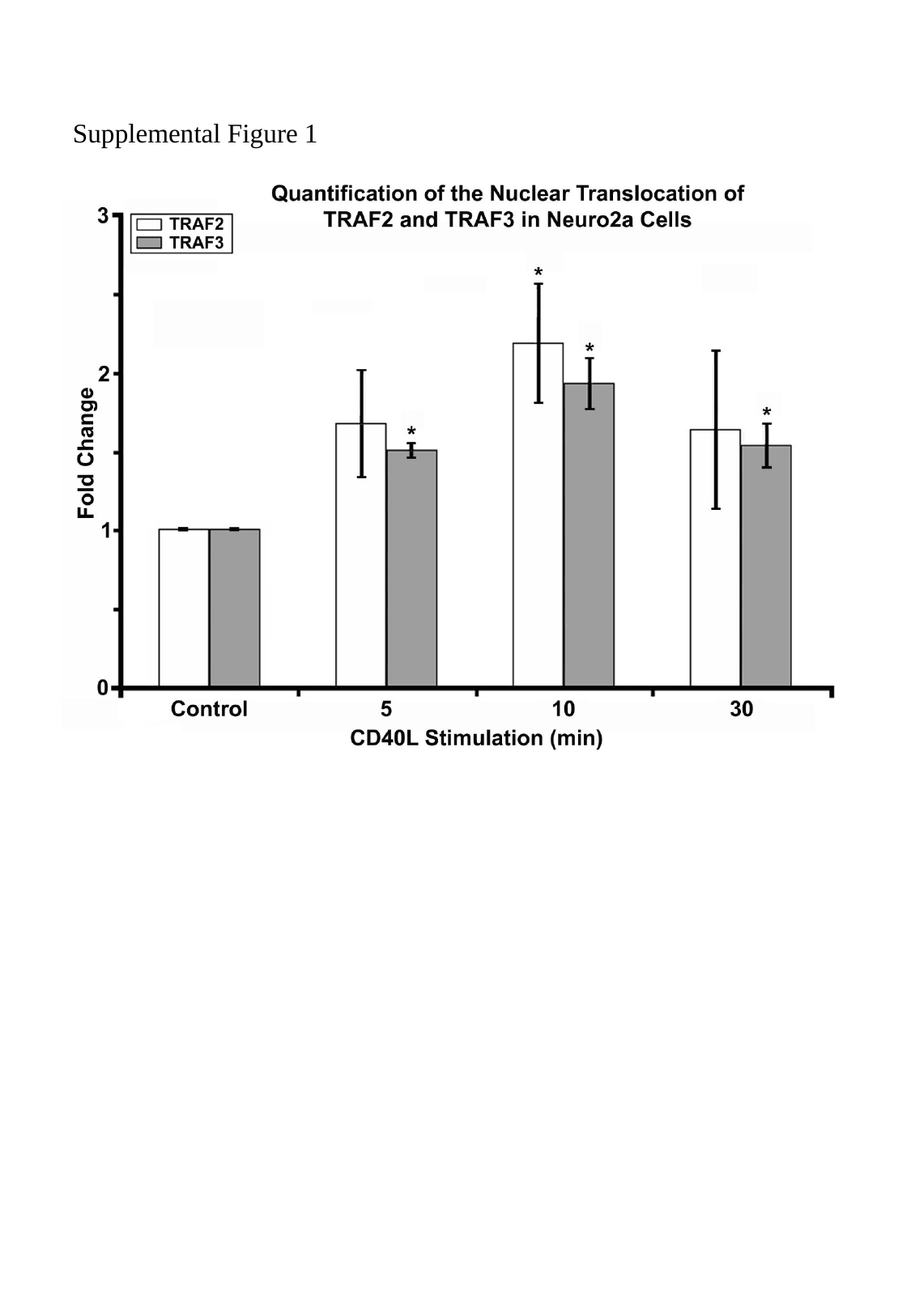

Supplemental Figure 1

Supplement: Supplementary file 1 — Quantification of the nuclear translocation of TRAF2 and TRAF3 in Neuro2a cells following CD40L-stimulation. The data represent the means of three independent experiments conducted in Fig. 2a +/- the SEM, quantified and normalized to Lamin A/C. One way ANOVA was performed, followed by Tukey’s test. * p ≤ 0.05. (PPTX 154 kb) [file 12035_2016_9742_MOESM1_ESM.pptx]

## Slide 1
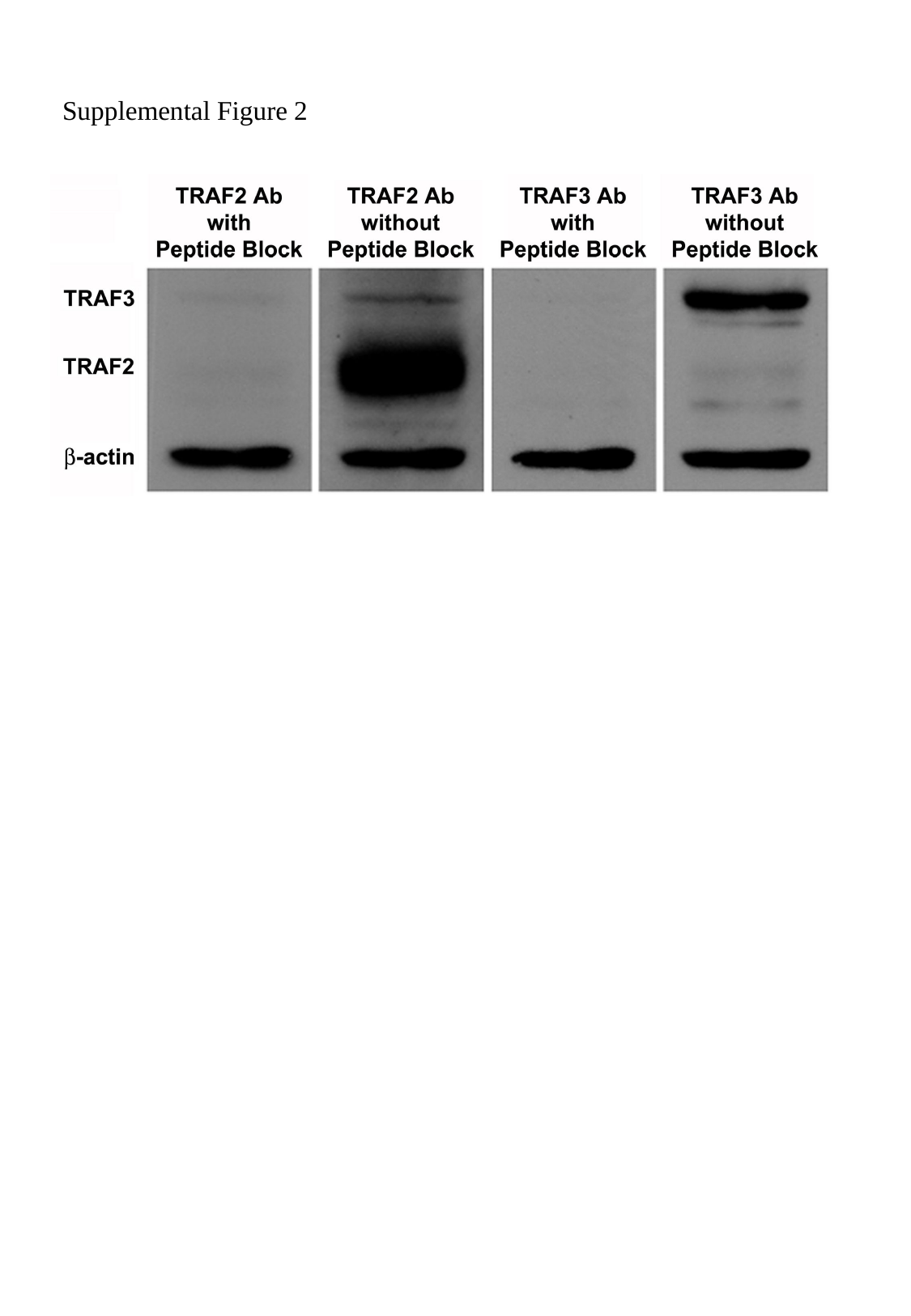

Supplemental Figure 2

Supplement: Supplementary file 2 — Verification of the specificity of the TRAF antibodies. A Western blot was performed with unstimulated Neuro2a whole cell extract (30 μg). The membrane was probed with an antibody to TRAF2 or TRAF3, either with or without preadsorption to their respective blocking peptide. Preadsorption was performed by incubating the antibodies at 4oC overnight with 10-fold excess blocking peptide. β-Actin was used as a control. Ab = antibody. (PPTX 403 kb) [file 12035_2016_9742_MOESM2_ESM.pptx]

## Slide 1
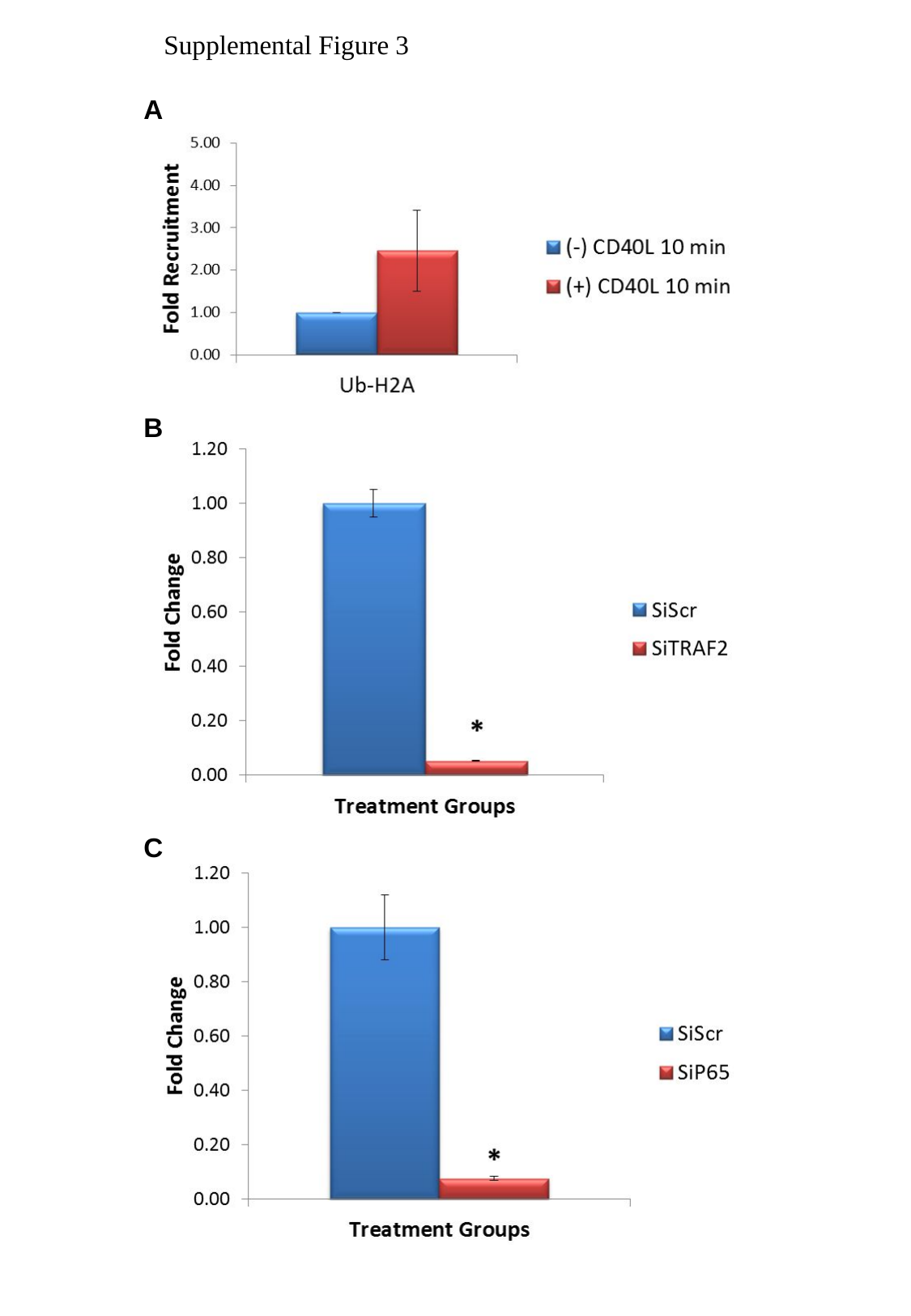

Supplemental Figure 3
A
B
C

Supplement: Supplementary file 3 — A) monoubiquitination of H2A upon CD40L stimulation. qPCR analysis of H2A-Ub (Lys 120) ChIP after 10 minutes of CD40L (100 ng/ml) treatment show insignificant H2A monoubiquitination at the p65 consensus sequence as compared to non treated cells. Non-specific IgG was used as an internal control while primers flanking an irrelevant control sequence were used as external controls. B) and C) Efficiency of TRAF2 and p65 knockdown in Neuro2a cells. qPCR analysis of TRAF2 (B) and p65 (C) mRNA expression show 95% and 93% knockdown efficiency respectively in Neuro2a cells as compared to Scrambled SiRNa (SiScr). (PPTX 117 kb) [file 12035_2016_9742_MOESM3_ESM.pptx]
